# Development and Feasibility of a Mobile Asthma App for Children and Their Caregivers: Mixed Methods Study

**DOI:** 10.2196/34509

**Published:** 2022-05-20

**Authors:** Misa Iio, Miori Sato, Masami Narita, Kiwako Yamamoto-Hanada, Taku Oishi, Ai Kishino, Takahiro Kawaguchi, Rin Nishi, Mayumi Nagata, Yukihiro Ohya

**Affiliations:** 1 College of Nursing Kanto Gakuin University Yokohama Japan; 2 Allergy Center National Center for Child Health and Development Setagaya Japan; 3 Department of Pediatrics, Kyorin University School of Medicine Kyorin University Mitaka Japan; 4 Department of Pediatrics, Kochi Medical School Kochi University Nankoku Japan; 5 Division of Pediatrics Tokyo Bay Urayasu Ichikawa Medical Center Urayasu Japan; 6 Division of Pediatrics Showa General Hospital Kodaira Japan; 7 Division of Pediatrics Yutenji Family Clinic Meguro Japan

**Keywords:** children, caregivers, asthma, mobile app, feasibility, health app, mHealth, pediatric, usability, mobile phone

## Abstract

**Background:**

Mobile health apps can support the self-management of pediatric asthma. Previous studies on mobile apps for children aged >7 years with asthma are limited, and most reports on asthma apps do not consider interactions between the children and their caregivers. Therefore, we developed an asthma app for children aged 0-12 years and their caregivers based on the results of our previous study regarding user needs.

**Objective:**

The aim of this study was to evaluate the feasibility of a developed mobile app for children with asthma and their caregivers and to modify and complete the app according to the feasibility results.

**Methods:**

We recruited children diagnosed with persistent asthma by an allergy specialist at 2 children’s hospitals, 1 university hospital, 2 general hospitals, and 1 pediatric clinic. Thereafter, the app usage was assessed, and questionnaires were administered. This study used convergent mixed methods, including providing user feedback about the pediatric asthma app, completing questionnaire surveys regarding preferences, and obtaining quantitative data about app usage. Quantitative data were analyzed based on the ratings provided for the app features used by the participants, and the usage of the app features was analyzed using descriptive statistics. Qualitative data were analyzed via a descriptive qualitative research analysis and were used to identify codes from the content-characteristic words.

**Results:**

In total, 30 pairs of children aged 2-12 years and their caregivers responded to the 3-month survey, and 20 pairs of children aged 4-12 years and their caregivers responded to the 6-month survey. In the 3- and 6-month surveys, “record” was the most commonly used feature by both caregivers and children. The average access logs per month among the 20 pairs ranged from 50 to 79 in the 6-month survey. The number of access logs decreased over time. In the qualitative results, app utilization difficulties were identified for 6 categories: record, preparing, alert settings, change settings, mobile phone owner, and display and motivation. Regarding app feasibility, 60% (12/20) of the caregivers strongly agreed or agreed for all evaluation items, while 63% (7/11) of the children strongly agreed or agreed for 6 items, excluding satisfaction. In the qualitative results, feasibility evaluation of the app was classified into 3 categories: high feasibility of the app, improvement points for the app, and personal factors preventing app utilization. Based on the results of the feasibility analysis, the final version of the app was modified and completed.

**Conclusions:**

The app feasibility among children with asthma and their caregivers was generally good. Children aged 7-12 years used elements such as record, quiz, and manga. This app can support the continuous self-management of pediatric asthma. However, efforts must be taken to maintain and improve the app quality.

**Trial Registration:**

UMIN Clinical Trials Registry UMIN000039058; https://tinyurl.com/3na9zyf8

## Introduction

### Background

Mobile health (mHealth) apps can support the self-management of chronic diseases, including medication use. Based on a previous review of digital health interventions for children with asthma, the implementation of such interventions may provide opportunity to improve treatment adherence and asthma control [1,2]. Digital health interventions for children with asthma include technologies that are useful for tracking asthma symptoms and medications, setting drug reminders, improving inhaler use techniques, providing asthma education, and using electronic monitoring devices, speech recognition calls, text messaging, mobile apps, and interactive websites [1].

mHealth apps for asthma generally target adult patients, and only few consider pediatric patients. Several mHealth asthma apps for children are intended for caregivers or adolescents who have their own smartphones. A systematic review of digital asthma self-management interventions was performed in the 2000s, and the interventions were commonly implemented among children aged 7-17 years [2,3] and their caregivers [4]. Since children aged >12 years usually have their own smartphones, previous studies on pediatric asthma apps usually included this age group [5,6]. Although digital self-management programs are available for children with asthma and their caregivers, only few studies have investigated the efficacy of these mobile apps.

Given the promising evidence of digital interventions for pediatric asthma, practitioners should support the use of evidence-based behavioral strategies to improve asthma management and health outcomes by recommending digital technologies and providing asthma education [2]. The features of asthma apps include asthma education material, symptom forecast, asthma action plan, telemedicine, local specialist connection, management during an emergency, symptom monitoring, airborne trigger identification, and clinic notifications [7]. The features of mHealth asthma apps are classified into 7 categories, that is, inform, instruct, record, display, guide, remind/alert, and communicate [8]. The most commonly used behavioral change techniques in asthma management apps are instruction, behavior-health association, self-monitoring, feedback, teach to use prompts/cues, consequences, and others’ approval [9]. An app can determine the asthma phenotype in children using the asthma control test by monitoring activity, sleep, peak expiratory flow, and indoor air quality [10,11].

Overall, there are only few apps that can be independently used for self-management by both children with asthma who are aged 7-12 years and 4-6 years and their caregivers. Most studies on asthma apps did not consider interactions between children and caregivers. Furthermore, previous studies on mobile apps associated with asthma self-management among children aged 7-12 years are limited. Therefore, we developed and improved a mobile asthma app for children and their caregivers based on the results of our previous survey [12], which assessed interactions between children and their caregivers. The proposed beneficial features of our app were identified under 6 themes: asthma knowledge, elements for continuous use, universal design, notification, monitoring, and functions [12].

### Development of a Mobile Asthma App for Children and Caregivers

We developed the prototype of a mobile asthma app for children and caregivers based on an individualized program using a touch-screen computer that we had developed [13]. The social cognitive theory [14] and tailoring [15] were used to develop a new mobile pediatric asthma app, which comprised a combination of asthma knowledge, behavioral change, and target behaviors and theory applications. The 3 main goals of this app are to (1) increase caregivers’ feelings of self-efficacy in asthma management, (2) improve asthma knowledge, and (3) facilitate continuous self-management behavior of controlling asthma symptoms.

**Figure 1:**
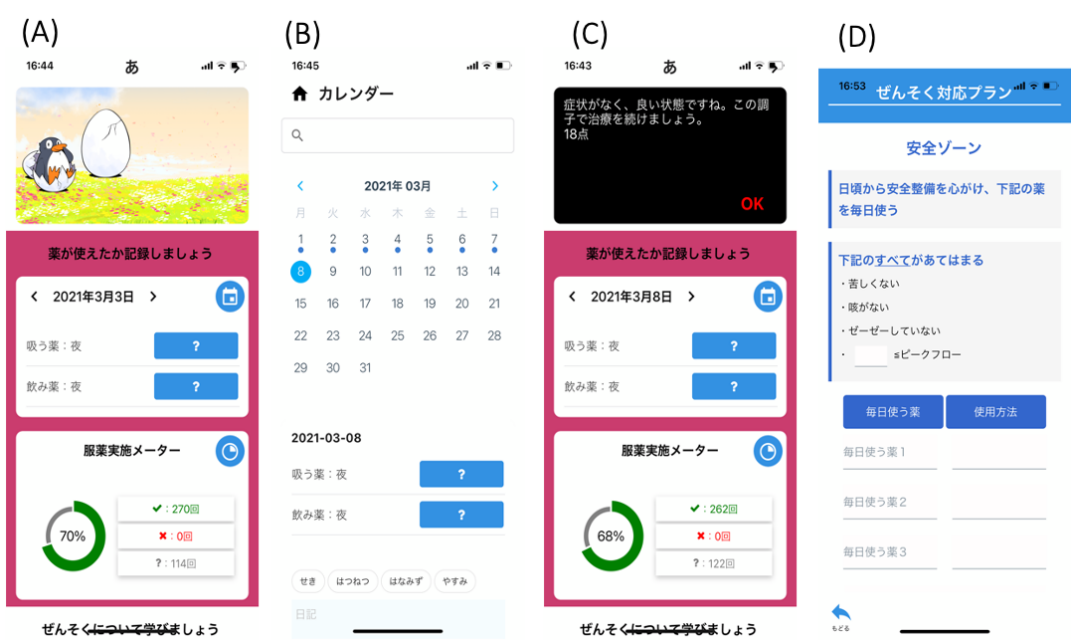
Examples of the different screens in the developed mobile asthma app for children and their caregivers. Examples of the screens commonly used by children aged <7 years and 7-12 years: (A) motivation screen and self-monitoring input of medications; the egg on the top panel grows after the continuous use of medications, and animals are born by breaking out from the eggs, (B) detailed calendar for self-monitoring, (C) tailored message display, and (D) asthma action plan for asthma exacerbation and disaster.

We improved and remade the prototype mobile asthma app for children and their caregivers based on the results of our previous survey [12]. In particular, the screen design of the app was changed to make it more user-friendly. The contents of self-monitoring of medication and symptoms were modified to separately classify medications and symptoms. In addition, a screen was added on the top page to motivate children to continue recording their medication behavior, that is, one can see an egg growing after the continuous use of medications, and animals are born after breaking out from the eggs. By maintaining a record of the medications, the egg cracks in 10 days, the animal inside it becomes slightly visible in 45 days, and finally, the egg hatches in 3 months. After the egg hatches, a new egg appears (Figure 1). Each egg has 6 animal characters, regardless of the gender of the child. Each time an egg hatches a character, a new egg appears.

**Figure 2:**
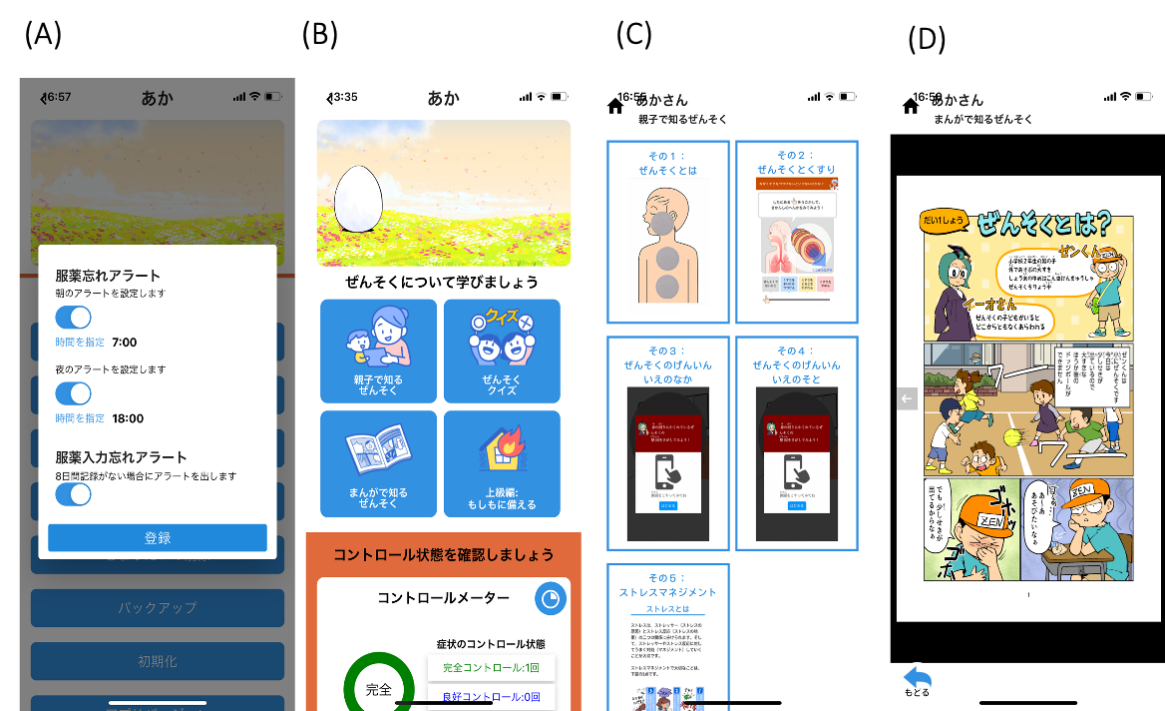
Examples of the different screens in the developed mobile child asthma app for children and their caregivers. Examples of the screens used by children aged <7 years and 7-12 years: (A) settings on the screen showing medication alert function, (B) screen for asthma knowledge and notification of the results in the pediatric asthma control test, (C) screen for asthma knowledge for children aged <7 years and caregivers, and (D) manga, a Japanese-style comic, for asthma knowledge for children aged 7-12 years and their caregivers.

This app was established for infants and toddlers (aged 0-6 years) and school-age children (aged 7-9 years and 10-12 years) according to their developmental stages. The common contents of the pediatric asthma app for children aged 0-12 years and their caregivers were self-monitoring of the medications and symptoms, preparing for asthma exacerbation and disasters, asthma action plan, and tailored feedback according to the Japanese pediatric asthma control test. Not only caregivers but also children could use the app based on their developmental stages. The common functions of the app for children aged 0-12 years and their caregivers were medication alert, app input alert, and notification of the results in the pediatric asthma control test (Figure 2). The individualized feedback messages based on the medication usage status were regularly displayed for the caregivers.

The contents of the asthma app for children aged 0-6 years and their caregivers focused on asthma knowledge (via a pictorial book about asthma clinical condition, causal factors, and complicating factors; quiz). These contents were mainly for caregivers, but the pictorial book could be completed by the child and the caregiver together (Figure 2). The pictorial book not only provides asthma knowledge but also promotes interaction between the child and the caregiver.

The contents of this app for children aged 7-12 years and their caregivers consisted of asthma knowledge (via manga, a Japanese-style comic, regarding asthma, medication, exercise-induced asthma, and stress management; quiz) and individualized feedback according to the status of medication usage. The difference in the app contents between children aged 7-9 years and 10-12 years was the use of “kanji,” a Japanese writing system that uses Chinese characters. Further, the app contents used manga, making the process of learning about asthma knowledge and management fun for children (Figure 2). Additionally, individualized feedback messages based on the medication usage status were regularly displayed for children and caregivers.

### Aim of This Study

Developing an evidence-based smartphone app requires conceptualization (understanding users’ needs and making decisions according to a theoretical basis) and pretesting feasibility and usability before efficacy evaluation [16,17]. To improve feasibility, we need to evaluate the performance of an app by allowing target populations to regularly use it. Therefore, this study aimed to evaluate the feasibility of a developed mobile app for children with asthma and their caregivers and to modify and complete the app according to the evaluation results. Evaluating the app’s feasibility can help improve the usage of mobile asthma apps among children and their caregivers. In addition, the mobile asthma app development must be completed by meeting the needs of the children and their caregivers.

## Methods

### Study Design

We used the convergent mixed methods design that included providing user feedbacks in a corrected pediatric asthma app, completing questionnaire surveys about preferences, and obtaining quantitative data about app usage. This study collected both qualitative and quantitative data, integrated both data, and derived interpretation from the combined strengths of both data. Compared with the use of 1 type of data, the integration of qualitative and quantitative data can provide a better understanding of the research subject. Therefore, this study design was selected because it was useful in evaluating the feasibility of the app.

### Recruitment

We recruited children aged 0-12 years who were diagnosed with persistent asthma by an allergy specialist at 2 children’s hospitals, 1 university hospital, 2 general hospitals, and 1 pediatric clinic. The details of persistent asthma, such as severity and treatment regimen and duration, were not considered. Considering the emphasis on the continuous use of the app for long-term medicine management, this study participants included children with persistent asthma, regardless of its severity. In Japan, children with asthma regularly visit hospitals or clinics based on the disease severity and complications of other allergic diseases. The target populations of this app are children with asthma and their caregivers, regardless of the hospital type they visit or the disease severity. Therefore, by recruiting children who visit various hospital types, the practicality of the app could be evaluated in a wide range of participants. Participants who were not eligible for this study owing to their mental and physical conditions based on the physician’s discretion were excluded. Purposive sampling, which can gather information-rich cases that manifest the phenomenon under investigation, was performed. Participants who met the inclusion criteria were selected based on data from the electronic medical records.

In this mixed methods study, we needed to consider the sample size options in both the quantitative and qualitative study stages. Our sample size options weighted the qualitative data to be equal to those of the quantitative data. The sample size was guided in line with consensus guidance [18]. For trade-offs between the breadth and depth of qualitative research, at least 30-60 participants is needed [19], given that less depth from a large number of people can be especially helpful in exploring a phenomenon and documenting diversity or understanding variation [18]. Therefore, researchers approached the caregivers of children with asthma who regularly visited the outpatient clinics. In total, 36 caregivers who met the inclusion criteria were contacted. The caregivers and children with asthma provided consent during the outpatient visit.

### Data Collection

We contacted 36 pairs of children with asthma and caregivers, of whom 34 agreed to participate in this study. Of the 36 caregivers who contacted us, only 1 caregiver of a child in the age group of 6-12 years did not participate because consent was not obtained. The other caregiver of a child in the age group of 0-6 years had difficulties in visiting the hospital regularly owing to moving to a different residence. The participants downloaded and used the pediatric asthma app for 6 months at home after providing consent. All participants answered the web-based questionnaire survey at 3 and 6 months from study registration. For children aged 0-6 years, only the caregivers answered the questionnaire, while both children aged 7-12 years and their caregivers answered the questionnaire. This study period was from June 2020 to March 2021. Data about the demographic characteristics of the participants, such as age, sex, age at asthma onset, and relationship with caregivers, were collected.

### Quantitative Data

Caregivers and children aged 7-12 years responded to the 3- and 6-month web-based survey questionnaires. After 3 and 6 months since app download, the app displayed an access link to the web-based questionnaire. After 6 months, we collected data about the number of access logs and the usage frequency of each feature (diary, asthma symptom control test, asthma knowledge: manga and quiz, preparing for asthma exacerbation, including asthma action plan, preparing for disaster, medication alert, and app input alert). The 3-month survey aimed to identify the usage frequency of the app features (diary, asthma symptom control test, asthma knowledge: manga and quiz, preparing for asthma exacerbation including asthma action plan, preparing for disaster, medication alert, and app input alert). Meanwhile, the 6-month survey collected data about the usage frequency of the app features, most commonly used app features, and app features that have never been used. To evaluate the specific elements of the app, we created a 7-item questionnaire according to the feasibility assessment using the 10-statement System Usability Scale by Brooke [20]. The participants rated feasibility on a 5-point scale for usability, benefit, satisfaction, continuous availability, intention of behavior, universal design, and view of tailored messages (1 [very bad] to 5 [very good]).

### Qualitative Data

After 3 months, the caregivers and children aged 7-12 years described the following 2 viewpoints in the web-based survey questionnaires: (1) barriers for the continuous use of the app and (2) facilitators for promoting the continuous use of the app. Additional demographic information such as age at asthma onset and treatment duration was collected. After 6 months, the caregivers and children aged 7-12 years provided their views about their impressions of the app. The 3- and 6-month web-based surveys were mainly used to identify the portion of the app that required final modifications and the proposed beneficial features of the app.

### Data Analysis

#### Quantitative Analysis

The questionnaire on the feasibility rating of the app features used and the usage frequency of the app features were analyzed using descriptive statistics. The number of access logs was summarized as the number of logs for each feature. Equivalence of demographic and medical information was compared using Fisher exact test at 3-month and 6-month time points. All statistical data were analyzed using the R software, version 3.6.1(R Foundation).

#### Qualitative Analysis

Qualitative data were transcribed verbatim in Japanese. A descriptive qualitative research analysis was performed to identify codes, subcategories, and categories from the qualitative data. Moreover, these data were analyzed in 2 phases: (1) becoming familiar with the collected data and (2) generating the codes and collating similar data for each code. Research members included 2 pediatric nurses (MI and MN) and 3 pediatricians who specialize in allergies (YM, KY, and MN). After the initial coding of each transcript, the researchers discussed and identified the categories and subcategories.

#### Integration

Quantitative and qualitative data were integrated, and a joint display was created.

### Ethics Consideration

This study was approved by the ethics committee for the Research of Social Medicine in the National Center for Child Health and Development (2019-103), the university ethics committee for studies involving human subjects, and the ethics committees for research involving human subjects in the 2 general hospitals. Participants who met the inclusion criteria and their caregivers were provided verbal and written information about the aim, significance, and methods of this study. Moreover, they were informed about their rights as voluntary participants, including study withdrawal, data anonymity, protection of confidential information, handling and disposal of data, and possibility of study publication. Participants aged 7-12 years provided written informed assent, and the caregivers of all the participants gave written consent.

## Results

### Characteristics of the Participants

Table 1 shows the characteristics of the participants. Of the 34 pairs enrolled in this study, 30 responded to the 3-month survey. Of those who responded to the 3-month survey, the average age of the children (16 boys and 14 girls) was 7.2 years and that of the parents was 42.4 years (27 mothers and 3 fathers). The average age of the infants and toddlers was 4.8 years and that of the school-age children was 8.9 years.

In total, 20 pairs responded to the 6-month survey; the average age of the children (12 boys and 8 girls) was 7.2 years and that of the parents was 41.9 years (17 mothers and 3 fathers). The average age of the infants and toddlers was 5 years and that of the school-age children was 9 years.

Of the 10 pairs that were lost during follow-up, 7 children were in the developmental stage of 7-12 years; these 10 pairs consisted of 6 girls and 4 boys, and all the caregivers were the children’s mothers. In the therapeutic regimen, 3 children used inhaled corticosteroids, 3 used leukotriene receptor antagonists, and 4 used inhaled corticosteroids+leukotriene receptor antagonists. Developmental stage, sex, therapeutic regimen, and caregivers’ relationship were not significantly different between the populations at the time points of 3 and 6 months.

**Table 1:** Characteristics of the participants who responded to the 3-month and 6-month surveys.

Demographic characteristics			3-month survey: 30 pairs (n)			6-month survey: 20 pairs (n)
**Children**						
	**Developmental stage (age)**					
		2-6 years		12	9	
		7-12 years		18	11	
	**Sex**					
		Boys		16	12	
		Girls		14	8	
	**Therapeutic regimen**					
		Inhaled corticosteroids		6	3	
		Leukotriene receptor antagonists		9	6	
		Inhaled corticosteroids+leukotriene receptor antagonists		15	11	
**Caregivers**						
	**Relationship to the children**					
		Mothers		27	17	
		Fathers		3	3	

### App Usage Status and the Main Codes in the 3-Month Survey

Table 2 shows the children and caregivers’ app usage status and the main codes in the 3-month survey. The most commonly used app features by the caregivers and children were “record,” that is, calendar/diary and asthma control check; the children most frequently utilized record. The total number of record access logs was 5153. The average access logs per month by the 30 pairs ranged from 45 to 69. About 61% (11/18) of the children used asthma manga. The usage rate of the asthma picture book among toddlers (n=12) and their caregivers was 25% (6/30). The app usage rate of preparing for asthma exacerbation and disaster was 27% (8/30) and 23% (7/30), respectively, and the lowest usage rate among children was 6% (1/18).

In the qualitative analysis results, difficulty in using the app contents was identified in 6 categories and 9 subcategories from 27 codes. These 6 categories were *record, preparing, alert settings, change settings, mobile phone owner, and display and motivation*. The coding and classifying phases revealed 3 subcategories in the category of *record*. According to the caregivers, “It can only enter about asthma” and “It takes time.” Both the caregivers and children reported that “It cannot record my daily physical condition, climate, and events.” The coding and classifying phases revealed 1 subcategory in the categories of *preparing*, and children claimed, “It does not know how to use preparing.” The coding and classifying phases revealed 1 subcategory in the categories of *alert setting*, and children mentioned, “It cannot display alert.” Another subcategory in the categories of *change setting* was noted to be difficult, and 1 child stated, “It does not know how to use the change settings.” The children answered “My mother was typing everything” and “The eggs do not grow, and the contents cannot be seen.”

Further, the coding and classifying phases revealed 1 subcategory in the categories of *mobile phone owner*. The caregiver and children reported “Not a child’s mobile phone.” Finally, another subcategory in the categories of *display and motivation* was revealed, and the caregivers and children responded, “The eggs do not grow and the contents cannot be seen.”

**Table 2:** Summary of the app usage status and categories of difficulties in using the app by children and caregivers in the 3-month survey.

App contents		Access logs (n)	Usage status of caregivers (n=30), n (%)	Usage status of school children (n=18), n (%)	Qualitative data Categories and subcategories of difficulties in using the app (number of codes)
**Record: medication and diary**		5153	24 (80)	11 (61)	**Record** It can only enter asthma. (Caregiver: 2 codes) It takes time. (Caregiver: 1 code) It cannot record my daily physical condition, climate, and events. (Caregiver and children: 9 codes)
	Average in the 1st month	69	—^a^	—	
	Average in the 2nd month	53	—	—	
	Average in the 3rd month	45	—	—	
Asthma control check		84	24 (80)	8 (44)	N/A^b^
Asthma picture book		20	6 (25)	—	N/A
Asthma manga		83	12 (40)	11 (61)	N/A
Asthma quiz		88	14 (47)	9 (50)	N/A
Preparing for asthma exacerbation		7	8 (27)	1 (6)	**Preparing** It does not know how to use *preparing*. (Children: 2 codes)
Preparing for disaster		2	7 (23)	1 (6)	
Setting of medication alert		52	14 (47)	2 (11)	**Alert settings** It cannot display alert. (Caregiver: 4 codes)
Setting of app input alert		—	8 (27)	2 (11)	
Tailored messages of medication		46	—	—	N/A
Tailored seasonal message		56	—	—	N/A
Change settings		21	—	—	**Change settings** It does not know how to use the change settings. (Children: 4 codes) **Mobile phone owner** Not a child’s mobile phone. (Caregiver and children: 3 codes) **Display and motivation** The eggs do not grow, and contents cannot be seen. (Caregiver and children: 2 codes)

^a^Not available.

^b^N/A: not applicable.

### App Usage Status and App Difficulty Categories in the 6-Month Survey

Table 3 depicts the children and caregivers’ app usage status in the 6-month survey. Record was the most commonly used featured among both caregivers and children. The total number of record access logs was 7628. The average access logs per month among the 20 pairs ranged from 50 to 79. Asthma control check was used by about 50% of both caregivers (9/20) and children (6/11). About 50% (6/11) of the children used the asthma manga and asthma quiz, and the rate was higher than that of caregivers. About 80% (24/30) of the participants used asthma control check, and the total number of access logs was 130.

**Table 3:** Summary of the app usage status of children and caregivers in the 6-month survey.

App contents			Access logs (n)		Frequently used app contents, n (%)				Most commonly used app content, n (%)				App content not used at all, n (%)		
					Caregivers (n=20)		Children (n=11)		Caregivers (n=20)		Children (n=11)		Caregivers (n=20)		Children (n=11)
**Record: medication and diary**			7628		15 (75)		9 (82)		10 (50)		9 (82)		2 (10)		2 (18)
	Average in the 1st month	79		—^a^		—		—		—		—		—	
	Average in the 2nd month	65		—		—		—		—		—		—	
	Average in the 3rd month	51		—		—		—		—		—		—	
	Average in the 4th month	50		—		—		—		—		—		—	
	Average in the 5th month	53		—		—		—		—		—		—	
	Average in the 6th month	52		—		—		—		—		—		—	
Asthma control check			130		9 (45)		6 (55)		2 (10)		2 (10)		1 (5)		—
Asthma picture book			16		2 (10)		—		2 (10)		—		1 (5)		—
Asthma manga			85		3 (15)		6 (55)		—		—		2 (10)		3 (27)
Asthma quiz			102		6 (30)		6 (55)		—		2 (18)		4 (20)		3 (27)
Preparing for asthma exacerbation			7		1 (5)		1 (9)		—		—		6 (30)		7 (64)
Action plan for asthma exacerbation			—		—		—		—		—		11 (55)		9 (82)
Preparing for disaster			1		2 (10)		1 (9)		—		—		5 (25)		7 (64)
Setting of medication alert			35		7 (35)		2 (18)		5 (25)		—		7 (35)		4 (36)
Setting of app input alert			—		3 (15)		2 (18)		1 (5)		—		8 (40)		4 (36)
Tailored messages of medication			88		—		—		—		—		—		—
Tailored seasonal message			90		—		—		—		—		—		—
Change settings			16		—		—		—		—		—		—

^a^Not available.

### App Feasibility Evaluation

**Figure 3:**
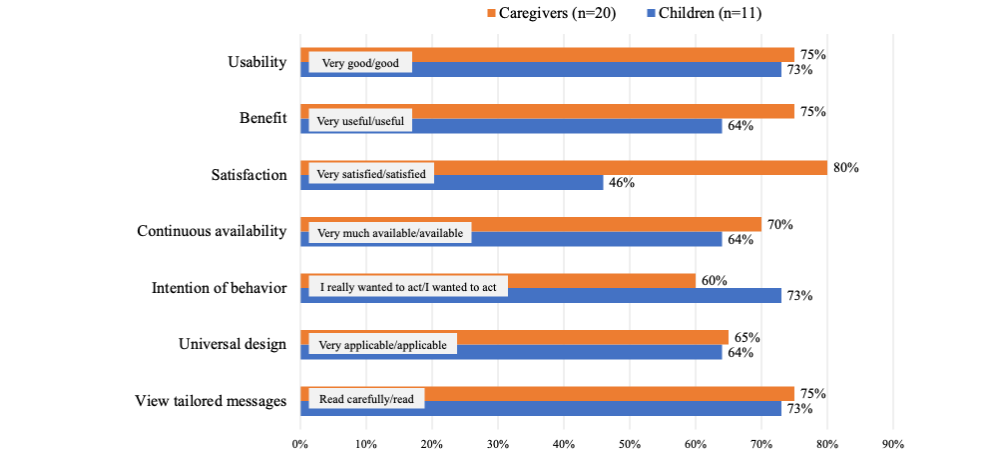
Evaluation of app feasibility. This graph shows the percentage of respondents who answered strongly agree and agree for the evaluation items.

Figure 3 and Tables 4 and 5 show the good- or low-feasibility evaluation results of the app among children and caregivers in the 6-month survey, that is, 70% (22/31) of the caregivers and children found the app usability to be very good or good. Several caregivers and children reported that “it was easy to use.” However, a caregiver reported that “entering the data took longer than I expected,” and another caregiver reported that “I was reluctant to let my child touch my smartphone.” About 80% (16/20) and 46% (5/11) of the caregivers and children were satisfied with the app, respectively. A caregiver stated that “I did not forget to take the medicine.” Meanwhile, both children and caregivers reported that “It was different from what I wanted to use.”

In the qualitative analysis results, good- or low-feasibility evaluation of the app was observed in 3 categories and 23 subcategories from 129 codes. By grouping the categories of good feasibility, a category called as *high feasibility of the app* was identified. This category comprised 11 subcategories: *simple, easy to use, convenience of alert, convenience of app, keeping records, opportunity to talk about asthma with a child, confirmation of asthma knowledge, action of management, user-friendly design, game elements,* and *existence value of app.* In addition, by grouping the categories of low feasibility, 2 categories, namely, *improvement points for app* and *personal factors in preventing to use of app* were identified. The category of *improvement points for app* comprised 9 subcategories: *not aware of contents of app, it takes time to input, hard to know how to use, understanding of child’s developmental stage, eggs do not break, design improvements, addition of motivational elements, gap with participants’ needs,* and *request for app.* The category of *personal factors in preventing to use of app* comprised 3 subcategories: *no time, can be managed without using app,* and *difficulty of children to use their caregivers’ mobile phone.*

About 60% (12/20) of the caregivers and 73% (8/11) of the children showed a positive intention of behavior in app usage. Caregivers reported “it was easy to manage because you always see your smartphone with the app” and “aiming for self-management by the child.” About 70% (14/20) of the caregivers and children read the tailored messages in the top page of the app. However, a caregiver reported that “I was too busy to read,” and a child answered “I did not know where the message was.” Overall, in terms of app feasibility, 60% (12/20) of the caregivers reported “strongly agree” or “agree” for all evaluation items, and 63% (7/11) of children reported “strongly agree” or “agree” for 6 items, excluding satisfaction.

**Table 4:** Good-feasibility evaluation of the app by caregivers and children.

Item		Caregivers (n=20), n (%)	Children (n=11), n (%)	Qualitative data Category and subcategories (number of codes)
**Usability**				**High feasibility of the app** Simple (Caregiver: 5 codes) Easy to use (Caregiver: 4 codes) Convenience of the alert (Caregiver: 5 codes) Convenience of the app (Caregiver: 4 codes) Keeping records (Caregiver: 12 codes) Opportunity to talk about asthma with a child (Caregiver: 2 codes) Confirmation of asthma knowledge (Caregiver: 6 codes) Action of management (Caregiver: 2 codes) User-friendliness (Caregiver: 4 codes) Game element (Caregiver: 4 codes) Existence value of the app (Caregiver: 4 codes)
	Very good	1 (5)	1 (9)	
	Good	14 (70)	7 (63)	
**Benefit**				
	Very useful	4 (20)	2 (18)	
	Useful	11 (55)	5 (45)	
**Satisfaction**				
	Very satisfied	2 (10)	0 (0)	
	Satisfied	14 (70)	5 (46)	
**Continuous availability**				
	Very much available	2 (10)	1 (9)	
	Available	12 (60)	6 (55)	
**Intention of behavior**				
	I really wanted to act	5 (25)	3 (27)	
	I wanted to act	7 (35)	5 (46)	
**Universal design**				
	Very applicable	3 (15)	1 (9)	
	Applicable	10 (50)	6 (55)	
**View tailored messages**				
	Read carefully	1 (5)	1 (9)	
	Read	14 (70)	7 (64)	

**Table 5:** Low-feasibility evaluation of the app by caregivers and children.

Item		Caregivers (n=20), n (%)	Children (n=11), n (%)	Qualitative data: Categories and subcategories (number of codes)
**Usability**				**Improvement points for app** Not aware of the app contents (Caregiver and children: 4 codes) It takes time to input (Caregiver: 6 codes) Hard to know how to use (Caregiver and children: 4 codes) Understanding of child’s developmental stage (Caregiver: 1 code) Eggs do not break (Caregiver and children: 7 codes) Design improvements (Caregiver: 7 codes) Addition of motivational elements (Caregiver and children: 6 codes) Gap with participants’ needs (Caregiver and children: 9 codes) Request for the app (Caregiver: 8 codes) **Personal factors in preventing app use** No time (Caregiver: 3 codes) Can be managed without using the app (Caregiver and children: 15 codes) Difficulty among children to use their caregivers’ mobile phone (Caregiver and children: 7 codes)
	Neither	3 (15)	1 (9)	
	Not very good	2 (10)	1 (9)	
	Very bad	0 (0)	1 (9)	
**Benefit**				
	Neither	3 (15)	1 (9)	
	Not very useful	1 (5)	3 (27)	
	Not useful at all	1 (5)	0 (0)	
**Satisfaction**				
	Neither	2 (10)	5 (46)	
	Not very satisfied	1 (5)	0 (0)	
	Not satisfied at all	1 (5)	1 (9)	
**Continuous availability**				
	Neither	3 (15)	2 (18)	
	Not very much available	1 (5)	1 (9)	
	Not available at all	2 (10)	1 (9)	
**Intention of behavior**				
	Neither	4 (20)	2 (18)	
	I did not want to act	4 (20)	1 (9)	
**Universal design**				
	Neither	3 (15)	3 (27)	
	Not very applicable	4 (20)	0 (0)	
	Not at all	0 (0)	1 (9)	
**View tailored messages**				
	I have not read much	5 (25)	2 (18)	
	I have not read at all	0 (0)	2 (18)	

### Final Version of the App

**Figure 4:**
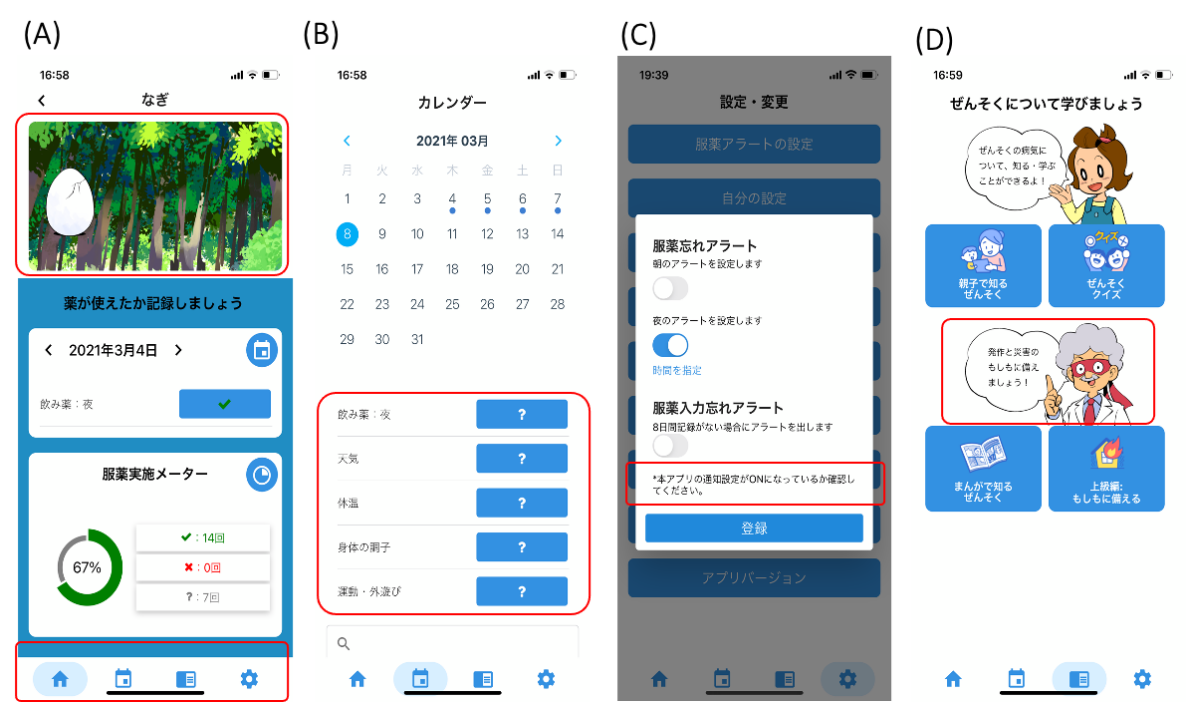
Modified contents of the mobile asthma app for children and their caregivers. The red frames indicate the commonly used options among children aged <7 years and 7-12 years, and added the tabs at the bottom of the top page. (A) Motivation screen and the self-monitoring input of medications, (B) detailed calendar for self-monitoring, (C) settings on the screen for medication alert function, and (D) asthma action plan in preparing for asthma exacerbation and disaster.

The final version of the app was modified and completed according to the access log, feasibility, usability, and qualitative feedback. We added tabs for easy operation, provided supplementary explanation of medication alerts, and added explanations to make the prepared version easily noticeable (Figure 4). By shortening the duration of egg breaking, the egg cracked in 5 days, the animal inside it became slightly visible in 20 days, and the egg finally hatched in 40 days.

## Discussion

### Principal Results

In this pediatric asthma app, the most commonly used feature of *records* by caregivers and children aged 7-12 years were medication and diary. The asthma manga and quiz features were more frequently used by school-age children than by caregivers. However, the app feature of preparing for asthma exacerbation and disaster was rarely used. About 50-70 access logs were observed per month among the 20 pairs of the participants. Regarding app feasibility, 60% (12/20) of the caregivers agreed in all the evaluation items, whereas 63% (7/11) of the children agreed in 6 items, excluding satisfaction. In the qualitative results, difficulties in using the app were identified under 6 categories: *record*, *preparing*, *alert settings*, *change settings*, *mobile phone owner*, and *display and motivation*. Additionally, app feasibility was analyzed under 3 categories: *high feasibility of the app*, *improvement points for app*, and *personal factors in preventing the use of the app*.

### Feasibility of the Asthma App for Children

This study aimed to complete the development of the pediatric asthma app according to the evaluation results of the app’s feasibility. The feasibility of the app was regarded as generally good by children with asthma and their caregivers. Elements such as record, quiz, and manga were utilized by children aged 7-12 years. Based on our previous study, children enjoy quiz and manga [12]. This study revealed that the access logs for manga were higher among children aged 7-12 years than in caregivers, indicating that manga was highly acclaimed by children with asthma in this age group. Meanwhile, these children and their caregivers reported that they used and accessed *record*, but children could not input their medication status because they did not have their own mobile phones. School-age children who did not have their own mobile phones found the app less feasible and useful than what their caregivers reported. Thus, the feasibility of the app according to children was influenced by mobile phone ownership.

For the continuous use of the app, we need to incorporate elements that capture children’s interest. Adolescents aged 11-18 years with asthma used the inhaler sensors of the mobile app with game features and reminders [21]. Moreover, they reported interest in the continuous use of the management system and would recommend this app to friends [21]. Although there is a difference that adolescents have their own mobile phone, elements such as games and comics that allow children to continue self-management with fun are important. However, in some cases, parents and children fight over the use of apps. This app intends to promote caregiver-child interaction. However, caregivers and children may have trouble using this app. Hence, it is necessary for them to discuss in advance how to use the app. In addition, the evaluation of app satisfaction showed that 80% (16/20) of the caregivers were satisfied or very satisfied. Meanwhile, only 46% (5/11) of the children aged 7-12 years felt the same. The satisfaction rating was also affected because children used the mobile phones of their caregivers instead of their own.

Asthma control check was the second most frequently used feature among children aged 7-12 years with asthma and their caregivers. In this study, we delivered tailored messages based on the results of the monthly asthma control test. However, we did not investigate the actual asthma control status for 6 months. Regarding technical feasibility, the usability score of the app was 78 based on the System Usability Scale, and approximately 75% (18/24) of the children with moderate-to-severe asthma indicated that eHealth helped them to control their asthma during the program [22]. A previous study [22] showed that eHealth care led to an 8.6% increase in asthma control, 25% in the self-management level, and 20.4% in therapy adherence. Therefore, the regular control tests and delivery of tailored messages may be effective in maintaining and improving asthma control.

Over time, the number of access logs decreased. A previous study has shown that the number of asthma app users decreases over time [23]. In this study, we instructed the participants to use the app only once a week or more on a regular basis. Thus, they could use the app freely. Of the 59 young adults with asthma who completed this study, 49 (83%) used the app ≥1 day per week [24]. Qualitative user experiences can be grouped under 2 themes: (1) learning how to use the app to suit the individual and (2) benefits and relevance of using the app [23]. When using the app, the content of the app must be used according to personal preference.

Adolescents identified various features such as ease of use and minimal effort as desirable criteria of an electronic monitoring device [24]. In terms of the usability evaluation in this study, 70% (22/31) of the caregivers and children reported good or very good. The results of the qualitative data indicated the high feasibility of the app, such as simple and easy to use. This app was generally easy to use. Hence, it is practical. The caregivers of the participants in this study emphasized the ease of using the app, good relationships with children, and support for self-management independence. In addition, caregivers captured the desire of children to use the app to understand asthma accurately and the mutual understanding of asthma with their children. In a previous study on a management system involving health care professionals, patients, and family members, family members supported patients in 269 (97.8%) of 275 coaching sessions [25]. That study showed that conversational agents, designed as mediating social actors involving health care professionals, patients, and family members, play not only the role of a “team player” but are also capable of improving health-relevant outcomes in chronic disease management [25]. The successful management of chronic diseases requires a trustful collaboration between health care professionals, patients, and family members. Asthma in children can be managed with the support of caregivers through the app.

As for the benefits of this app, 75% (15/20) of the caregivers reported the app to be useful or very useful. Children and adolescents aged 8-17 years and their caregivers preferred the use of technology to facilitate medication and disease management, and children had a strong willingness and ability to actively engage in their care [26]. A previous study [27] revealed that 14 children and adolescents aged 8-17 years and their caregivers reported the acceptability of using smartphones for real-time asthma monitoring. The app was easy and practical for caregivers and children. The app wireframe for adolescents based on the self-regulation theory was generally well-received, and suggestions on how to improve the app included further customization of charts and notifications, reminders, and alerts [28]. The participants believed that the app was generally useful for managing their asthma. This feasibility study revealed areas for improvement for the asthma app for children aged 0-12 years and their caregivers. Specific improvements based on the subcategories shown in the qualitative analysis results were indicated. Addressing these areas is important in developing an engaging and effective pediatric asthma app.

An interactive digital solution study showed the feasibility and benefits of deploying user-centric design methods that engage real patients and caregivers throughout the health technology design process [29]. Another study proposed a paradigm shift—from providing features that are easy to implement technologically to using an approach in which apps are designed to deliver theoretically grounded preferred components [30]. This app was developed and modified based on theoretically grounded preferred components of our previous studies [12,13]. Moreover, it reflected the opinions of health care professionals, pediatric patients with asthma, and caregivers during the app development process. Based on a previous review about the potential of publicly available and well-adopted mHealth apps for improving asthma self-management, the apps can be consistent across review frameworks [31]. However, several apps had low quality [32]. This app can support continuous self-management of pediatric asthma. However, efforts must be taken to maintain and improve the app quality.

### Limitations

This study had several limitations. The 3-month survey findings of the 30 pairs of participants and the 6-month survey findings of the 20 pairs of participants were used to identify the feasibility of the app among children with asthma and their caregivers. However, although we recruited 34 pairs of participants in this study, 14 pairs dropped out. The web-based survey showed a survey link in the app at 3 and 6 months after the app was installed. As a factor of dropout, the participants had to answer on the spot, and once the participants close the survey form, the questionnaire could not be accessed again; therefore, we could not consider the 14 pairs who dropped out. However, the evaluation of the 14 pairs with regard to app feasibility would have provided potentially more information.

The caregivers were usually mothers. Although the number of double-income families is increasing in Japan, mothers still attend the outpatient visits of children. Further, children with persistent asthma were recruited in different types of hospitals such as children’s hospital, university hospital, general hospital, and clinics, but this was not considered as a potential selection bias.

The severity of persistent asthma was not taken into consideration during participant selection. The app could be used by any child with asthma anytime and anywhere in Japan. Therefore, the results were useful in evaluating the app feasibility in the target population: children aged <7 years and 7-12 years with asthma and their caregivers. In addition, this study validated the caregiver-reported feasibility of the app among children aged <7 years. However, children aged <7 years had difficulties in answering the questions accurately, given the limited language function and cognitive development. Therefore, caregivers were instructed to provide information regarding their needs.

The feasibility of the app was evaluated using a quantitative questionnaire that was developed by us, according to the computer system usability scale [20]. This questionnaire is not yet validated and adopted by other studies, thereby lacking external validity.

The mobile phones of the caregivers of children aged 7-12 years were used, as they do not have their own phones. This study showed inconveniences in the children’s inability to use the phones freely and in their efforts toward self-care independence. However, using a caregiver’s mobile phone can lead to communication and interaction between children and their caregivers.

### Strengths and Future Research

The strengths of this study are as follows. This study included preschool children aged 0-6 years and 7-12 years who were not previously targeted by app developers. The data collected from children aged 2-12 years, such as usage of data app features and their comments regarding the app, could help evaluate its feasibility and operability. In addition, it incorporated eggs that grow with the continuous use of medications and typing, which could have motivated the children with asthma and their caregivers.

The efficacy evaluation of the app will help practice effective patient education among preschool and school-age children (0-12 years) with asthma and their caregivers. In the future, the efficacy of the contents of the final version of the app must be evaluated. In addition, the behavior associated with the app usage should be considered.

### Conclusions

The pediatric asthma app feasibility among children with asthma and their caregivers was generally good. Children aged 7-12 years had used elements such as record, quiz, and manga. Based on the usage data of the app features and the comments regarding the app among children aged 2-12 years with asthma and their caregivers, the app generally had good feasibility and operability. Hence, this app can support the continuous self-management of pediatric asthma. Nevertheless, efforts must be taken to maintain and improve the app quality.

## Abbreviations

mHealth: mobile health
